# Multi-Way Multi-Group Segregation and Diversity Indices

**DOI:** 10.1371/journal.pone.0010912

**Published:** 2010-06-01

**Authors:** Root Gorelick, Susan M. Bertram

**Affiliations:** 1 Department of Biology, Carleton University, Ottawa, Canada; 2 School of Mathematics and Statistics, Carleton University, Ottawa, Canada; University of Bristol, United Kingdom

## Abstract

**Background:**

How can we compute a segregation or diversity index from a three-way or multi-way contingency table, where each variable can take on an arbitrary finite number of values and where the index takes values between zero and one? Previous methods only exist for two-way contingency tables or dichotomous variables. A prototypical three-way case is the segregation index of a set of industries or departments given multiple explanatory variables of both sex and race. This can be further extended to other variables, such as disability, number of years of education, and former military service.

**Methodology/Principal Findings:**

We extend existing segregation indices based on Euclidean distance (square of coefficient of variation) and Boltzmann/Shannon/Theil index from two-way to multi-way contingency tables by including multiple summations. We provide several biological applications, such as indices for age polyethism and linkage disequilibrium. We also provide a new heuristic conceptualization of entropy-based indices. Higher order association measures are often independent of lower order ones, hence an overall segregation or diversity index should be the arithmetic mean of the normalized association measures at all orders. These methods are applicable when individuals self-identify as multiple races or even multiple sexes and when individuals work part-time in multiple industries.

**Conclusions/Significance:**

The policy implications of this work are enormous, allowing people to rigorously test whether employment or biological diversity has changed.

## Introduction

There exists a much-deserved impetus to increase employment of traditionally under-represented groups. We need to do more than increase the number of sexes and races represented across each department, organization, or industry. Presence/absence data of traditionally under-represented groups should not be the benchmark. Given two industries that hire men and women of all locally-recognized racial groups, how do we determine which has greater employment diversity or segregation? How do we ensure that industries, such as universities, are not just reaching a token form of diversity, where most women and people of color are hired into women's studies, ethnic studies, and social science departments, but where they are absent from engineering, mathematics, and physical science departments? What measure of diversity will detect white women being exclusively hired in clerical positions, people of colour being hired in custodial positions, and white males being hired in white-collar positions? A robust diversity statistic would have to measure whether traditionally under-represented groups have an equal footing in all departments and all job descriptions, what ecologists call *β*-diversity [1], [2]. Quantification should allow comparison of an industry's employment diversity or segregation against itself at an earlier time, or against another industry at the same time.

Similarly, biologists want to preserve diversity, but not just the number of species over geographic landscapes. Other explanatory variables can include age class of individuals, their sex, or health.

Sociologists usually measure segregation. Biologists see the flip side of the coin and measure diversity. Diversity and segregation are really the same entity, which sociologists clearly realized in using the term ‘ecological segregation’ to refer to racial segregation [3], [4]. When indices range from zero to one, diversity is simply one minus segregation. Both biologists and sociologists have a real need to compute diversity/segregation indices from multi-way contingency tables of variables that can take multiple values. While we can discuss segregation and diversity in the same breath, the state-of-the-art seems better developed in sociology than in biology. We will therefore focus largely on the sociological nomenclature, discussing several biological applications at the end.

Given a contingency table of categorical variables, how do we compute a scalar segregation index, for which a value of zero reflects no segregation and non-zero values are proportional to amount of segregation? The other way to look at this is via one minus this index, for which a value of zero reflects zero diversity and non-zero values are proportional to amount of diversity. Early work focused on measuring segregation of one binary (dichotomous) category, such as female versus male or white versus non-white, against a second multi-group categorical variable, such as industry, company, or department [5], [6], [7]. More recent work has focused on quantifying segregation/diversity when both variables are multi-group, such as race that can take on a countable number of values [8]. However, there do not appear to be any segregation indices that include multiple multi-group explanatory variables. Yet, there should be a huge impetus to measure such multi-way multi-group segregation indices, such as an outcome variable of industry and several simultaneous explanatory variables, possibly including race, sex, number of years of education veteran's status, presence of disabilities, etc. The data clearly exist [9]; only the methods are lacking.

Before delving into computation of segregation/diversity indices, we would be remiss to not mention a parallel set of developments, such as the log-linear models for analyzing contingency tables, including those between more than two multi-group variables [10]. While these methods allow for hypothesis testing of independence, they do not provide a single scalar measure of segregation or diversity. Instead, these other methods attempt to explain how segregation varies between categories by, say, producing a graph of segregation versus category [11], [12]. We are not disparaging of these excellent methods, but would rather delve into the ‘index wars’ [13] or, more accurately, to extend existing index computations.

Sociologists have also created inequality indices, which measure how well each class of workers does in the labour market, examining things such as glass ceilings [14]. While important, inequality indices help in answering a different set of questions than what is the total level of segregation or diversity of sexes and races across all industries, organizations, or departments.

Many of the above measures of segregation are normative, i.e. they describe what ought to be, such as equality in pay or employment across races, sexes, or other categories. For ecological examples, the normative question might be: How close to ‘optimum’ *β*-diversity is a given population? Segregation indices that range between zero and one can be used in a normative fashion, such as striving for segregation index values of zero (high diversity) in a population. Such segregation indices can also be used in a positive fashion, such as discerning which of two industries has smaller segregation (greater diversity) or whether a given industry has had a decrease in segregation (increase in diversity) over time. Positive applications of these indices are sometimes known as empirical measures of segregation, equality, or inequality [15]. In biology, positive approaches would be to ask which of two populations had greater *β*-diversity, had greater age polyethism, or had greater linkage disequilibrium. We take the positive/empirical perspective on segregation/diversity indices, although admit that they can form a basis for a more normative approach.

## Methods

### Reardon & Firebaugh's two-way multi-group association-based segregation indices

Reardon & Firebaugh [8] provide a marvelously simple and sensible framework for constructing segregation indices based on association measures. Their association measures – which are un-normalized segregation indices – are the weighted means of some function (to be specified) of the ratio of observed-to-expected values of each entry in a contingency table, where the mean is weighted by the expected values. This class of association measures is determined by (1) a model for the expected value of each cell in the contingency table and (2) the function of the ratio of observed-to-expected. The beauty of their approach is that such measures can be extended from two-way to multi-way contingency tables, even though Reardon & Firebaugh (2002) never stated this. Their formula for the weighted mean is 
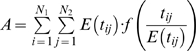
, where *E* stands for expected value (which depends on our model of the world), 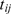 is the contingency table value for race *j*, industry *i*, and *f* is a continuous function for which 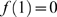, i.e. *f* yields small values when observed is approximately equal to expected. Our primary contribution is to extend Reardon & Firebaugh's formula for *A* to a multi-way contingency table. For example, for a three-way contingency table, how do you compute the square of coefficient of variation? We will show that this also requires examining *A* for projections (marginals) of the original contingency table.

To make the above methodology operational, we have to specify a model for the expected value of each term in the contingency table and the weighting for the mean. For the expected value, most authors use independence, i.e. joint probability (which is the value in the contingency table) equals the product of the marginal probabilities (which are the probabilities of the rows and columns). While we adopt this model for expected value, alternative models could be used. Regarding choice of weighting for the mean, Reardon & Firebaugh [8] provide two functional forms: an l^2^ (aka: euclidean) norm to measure distance from unity, 
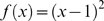
, and the Boltzmann/Shannon/Theil form of 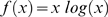 [16], [17], [18]. Later, we mention how other functional forms are possible. Finally, Reardon & Firebaugh normalize association measures (*A*) so that they yield values between zero and one, which they do by dividing by the maximum possible value. We follow this standard approach to normalization.

### Minor variations on Reardon & Firebaugh

To compute segregation indices via association measures, consider an 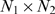 (two-way) contingency table, with say 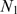 industries and 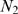 races. Entries in the table represent the number of members of each race in each industry or department. Although not highlighted by others, this framework allows for multi-racial individuals, where each individual is counted once. If an individual self-identifies as being in three races, with half of the identity being in one race and a quarter of their identity being in two other races, then split them accordingly. Likewise, if people work half-time in two different industries, then we can count them as participating as a half-person in each industry. We could even have people self-identify in both sexes.

There are many segregation indices based on association measures, many of which are slight modifications of existing formulae [19]. However, quoting Duncan & Duncan's [20] seminal paper, Reardon & Firebaugh [8] admonish everyone to ground segregation indices in sound theory. The functional forms 
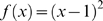
 and 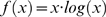 fit that bill, at least if we first convert the observed and expected values in the contingency table to probabilities, in which all entries sum to one: 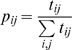.

The expected value, 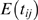, which has been converted to 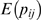, can take many forms. For instance, the number of workers expected per industry might be equal for each race and for each sex. Or, the expected number of workers per race might be proportional to the number of people belonging to each race [3], [4], using census data. The most parsimonious model is of independence [10]. More precisely, 
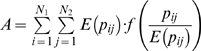
 and row and column sums are 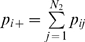 and 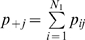. Then independence implies that the expected value is 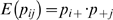, so that 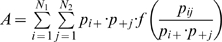, a convention that we use throughout the remainder of this paper, but an assumption that can be relaxed.

Having converted to probabilities and assumed independence, 
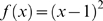
 yields a weighted mean that equals the square of the coefficient of variation, whereas 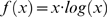 yields a weighted mean that equals Shannon's mutual entropy. Both coefficient of variation and mutual entropy have rich theories, thereby justifying the functional forms that generated them. Other functional forms might also make sense. Instead of an l^2^-norm, one could invoke an l^1^- norm, i.e. 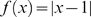. Gorelick & Bertram [19] discuss these different choices of norm and when they have been used, especially in the work of Smith & Snow [21]. Other logical possibilities are 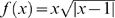 and 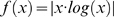 In some ways, 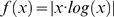 makes more sense than 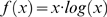 because antithetically the latter takes on negative values when *x* is less than one, although we have never seen the functional form 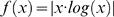 used. Regardless, as we show in the next paragraph, there are good reasons to use 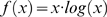.

How do we heuristically conceptualize segregation? Consider the example of a university that is divided into several departments. Which university department do you suspect has a greater proportion of men (or women), physics or women's studies? If you guessed more men are typically faculty members in physics and more women are typically faculty members in women's studies, you would undoubtedly be correct because these departments are notoriously segregated. With high gender segregation, given a department, we can readily predict the sex of faculty members. Likewise, with high gender segregation, given the sex of a faculty member, we can readily predict a subset of departments to which they probably belong. Conversely, if there is virtually no gender segregation, given a department, we cannot readily predict the sex of the faculty member. Without gender segregation, given the sex of a faculty member, we also cannot readily predict their department. Segregation, therefore, is directly proportional to what any given dimension of the array tells us about the other dimensions. If we focus on just departments and race for a moment, this translates into the following. High racial segregation means that rows of the matrix (departments) convey lots of information about the columns of the matrix (race), and vice versa. This conceptualization of diversity is encapsulated by mutual entropy, in which Shannon [17] measured how much information was transmitted back and forth between sender (rows) and receiver (columns). Mutual entropy is directly proportional to segregation. Thus 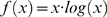 is a natural choice for generating an association based segregation measure.

### Major variations on Reardon & Firebaugh

We extend Reardon & Firebaugh's [8] segregation indices in two substantive ways. First, we extend their two-way multi-group indices to multi-way multi-group indices. Second, when extending to multi-way indices, not only is the highest order index important, but the lower order indices – which are projections or aggregates of variables – also provide useful measures of segregation. Therefore, we propose computing the arithmetic mean of normalized segregation indices of all orders, which can be readily converted to a diversity index.

The beauty in defining 
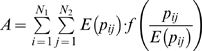
 with various forms for *f* is that this can easily be extended to multiple input variables, such as an 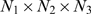 contingency table because of our simple multiplicative definition of independence. Define the marginal probabilities as 
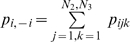
 or more simply written 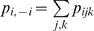. That is, for any value of *i* (which could be industries) add up over all values of *j* and *k* (which could be races and sexes). Due to independence, 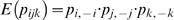, a definition that can easily be extended to more than three dimensions. This works with any of the forms of 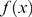. For example, the Boltzmann/Shannon/Theil segregation index becomes the multi-dimensional form of mutual entropy [22], [23].

Computing the highest dimensional association measure does not, however, provide all the clues needed for quantifying segregation. Consider *B* through *G* below, for which we apply Boltzmann/Shannon/Theil association measure 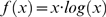. These scenarios each have two industries, two races, and two sexes. Scenario *B* has industry 1 with one-quarter of the population as black females and another quarter as white males. Industry 2 has one-quarter of the population as white females and the last quarter black males. The slices of the data array for the two industries are:
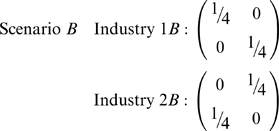
Scenario *C* has both industries that are identical to industry 1 in scenario *B*. That is, regardless of industry, only black females and white males are employed. All white females and black males are unemployed.
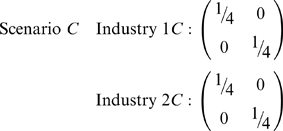
Clearly there is more segregation (less diversity) with scenario *C*, yet the association measure incorporating all three dimensions – industry, race, and sex – is the same: 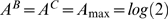. We briefly digress and show how to compute 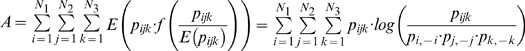 for scenario *B* given 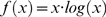 and independence. Half of the eight elements, 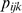, in this three-dimensional contingency table equal zero and the other half equal one-quarter. The row and column sums all equal one-half, i.e. 
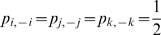
. Thus 
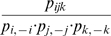
 either equals 0 or 2. In the former instance, 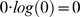, per l'Hôpital's rule 
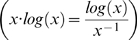
. Putting this all together, 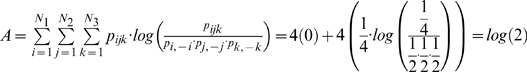.

The key to distinguishing scenarios *B* and *C* is to not just examine three-way interactions of industry x race x sex, but also to examine the projections along each of the axes. For instance, aggregating data across industries:
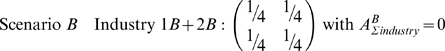


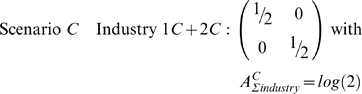
Aggregating (aka ‘collapsing’ or ‘projecting’) a variable determines the source of segregation. In scenario *C*, there is no diversity between industries (1*C* = 2*C*) and correspondingly there is maximal segregation as seen via 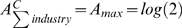. If instead we aggregated race or sex, then 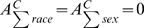, indicating that segregation is not *per se* simply due to race or sex, but rather to interactions of industry with race and/or sex.

Likewise, it is possible to have this same level of overall segregation, 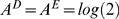, if both industries segregate based on race, but not sex…or vice versa:
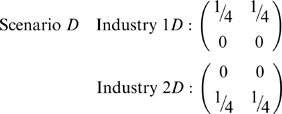


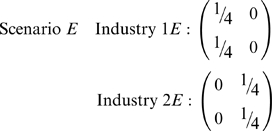
To distinguish these from scenario *B*, aggregate/project contingency tables along the directions of race and sex. For scenarios *B* and *C*, 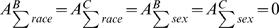. For scenario *D*, 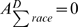 and 
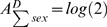
, whereas for scenario *E*, 
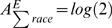
 and 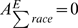.

It is also crucial to aggregate/project the 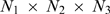 contingency table in pairs of directions, as can be seen with the following pair of scenarios:
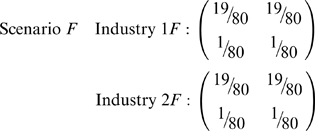


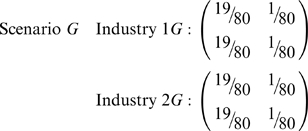
Scenarios *F* and *G* both have 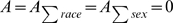, despite clear segregation by race or sex, respectively, in *F* and *G*. Consequently, also compute 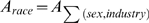 and 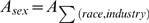, which here are merely the Boltzmann/Shannon/Theil index of vectors, more commonly denoted 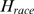 and 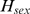.

Segregation indices are normalized to be between zero and one by dividing an association measure by its maximum value, 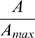. For any sensible association measure, 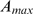 occurs when all entries of the contingency table are equal to one another. For the Boltzmann/Shannon/Theil index and an *M*-dimensional contingency table 
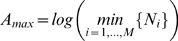
. For a three-way contingency table comprised of industry, race, and sex, it is therefore imperative to also compute 
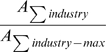
, 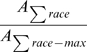, 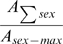, 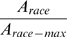, and 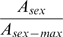. Each of these association indices on two-way contingency tables – 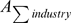, 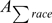, 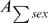, 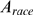, 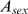, which have been projected from a three-way table, – are simply the old-fashioned Theil's index or entropy. Projections along any pair of axes are the marginal of the remaining axis, i.e. 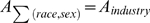 or Shannon's index on a vector.

Our main objective is to produce a segregation index that reflects segregation at any level. Therefore compute the arithmetic mean of all orders of segregation. The overall segregation index, 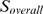, for a three-way contingency table of industry, race, and sex is thus 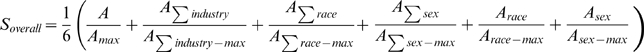. This overall segregation index will be between zero and one because it is a mean of numbers that all lie between zero and one. Table 1 provides several examples of segregation/diversity of industries in which there are two departments, two races, and two sexes (using scenarios *B-G* from above). While computationally uglier, the above formula for overall segregation can easily be extended to multi-way contingency tables of any dimension – there are simply many more terms because there are many different ways to project higher-dimensional contingency tables.

**Table 1:** Seven examples of overall segregation index computed using the Boltzmann/Shannon/Theil association measure, with expected values based on independence.

Example	Department	Sex	Majority	Minority	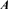	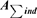	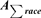	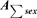	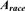	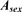	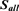
**A**	Sciences	Male	100	100							
		Female	100	100	0	0	0	0	0.699	0.699	
	Humanities	Male	100	100	(0)	(0)	(0)	(0)	(0)	(0)	**0**
		Female	100	100							
**B**	Sciences	Male	200	0							
		Female	0	200	1.000	0	0	0	0.699	0.699	
	Humanities	Male	0	200	(1.00)	(0)	(0)	(0)	(0)	(0)	**0.17**
		Female	200	0							
**C**	Sciences	Male	200	0							
		Female	0	200	1.000	1.000	0	0	0.699	0.699	
	Humanities	Male	200	0	(1.00)	(1.00)	(0)	(0)	(0)	(0)	**0.33**
		Female	0	200							
**D**	Sciences	Male	200	200							
		Female	0	0	1.000	0	1.000	0	0.699	0.699	
	Humanities	Male	0	0	(1.00)	(0)	(1.00)	(0)	(0)	(0)	**0.33**
		Female	200	200							
**E**	Sciences	Male	200	0							
		Female	200	0	1.000	0	0	1.000	0.699	0.699	
	Humanities	Male	0	200	(1.00)	(0)	(0)	(1.00)	(0)	(0)	**0.33**
		Female	0	200							
**F**	Sciences	Male	190	190							
		Female	10	10	0	0	0	0	0.699	0.914	
	Humanities	Male	190	190	(0)	(0)	(0)	(0)	(0)	(0.71)	**0.12**
		Female	10	10							
**G**	Sciences	Male	190	10							
		Female	190	10	0	0	0	0	0.914	0.699	
	Humanities	Male	190	10	(0)	(0)	(0)	(0)	(0.71)	(0)	**0.12**
		Female	190	10							

The subscript *industry* is abbreviated *ind* and *overall* is abbreviated *all*. For each scenario, the first row of values are the association measures, while the second row (in parentheses) are association measures divided by their respective values of 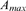. Note that scenario A provides the calculation of these maximum values. Summations in subscripts refer to aggregations/projections along one or more dimensions of each array. Furthermore, 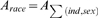, which is the association measure of the aggregation/projection across the dimensions of *industry* and *sex*, i.e. all the dimensions other than *race*. Likewise, 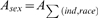 is the association measure after collapsing all dimensions other than *sex*.

Diversity is the flip side of segregation: 
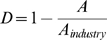
, 
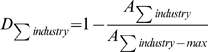
, 
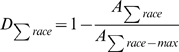
, 
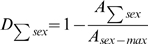
, 
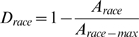
, 
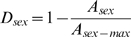
, and consequently 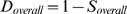.

## Results

Not applicable.

## Discussion

### Biological applications

The examples given above have been entirely from sociology, yet the same methods are applicable to many biological questions. We briefly outline how association measures of segregation and their flip side of diversity can be useful in biology.

Ecologists often wish to compare biological diversity between two different parcels of land or changes in diversity over time in a single parcel. This is usually conceptualized as a two-way multi-group contingency table, where the rows of the table are species, the columns are subplots of land inside the large parcel, and numbers in the table are species abundances per subplot. Standard association measures are applied, such as mutual entropy or coefficient of variation, to obtain total biological diversity over the parcel. We can now include additional independent variables, such as the sex or age-classes of individuals of each species. Conservation biologists could use sex to gauge the potential for local extinction, whereas foresters could use age classes for gauging health of wooded parcels.

Biologists also are interested in segregation, especially in animal behaviour. For example, what determines mate choice [24]? For each female in a population (the dependent or outcome variable), we can ask how likely she is to mate with males that possess certain independent characters, such as number of brightly coloured spots and long tail feathers. Association measures thereby provide a measure of assortative mating.

We had earlier described segregation as division of labour [25], where the two-way contingency table contained individuals and tasks. This becomes a three-way contingency table with the addition of age of each individual and association measures then yield measures of age polyethism.

Linkage disequilibrium is easy to measure for two genetic loci (a two-way contingency table), but is conspicuously complicated for multiple genetic loci [26], [27]. However, an alternative approach is to compute linkage disequilibrium as an association measure of the contingency table [28], which can be easily extended to multiple loci using the methods herein [29].

### Concluding remarks

Previously, indices of segregation and its mirror-image diversity had been quantified for two-way multi-group contingency tables, but not for multi-way tables. These two-way indices have proven extremely valuable in both social and natural sciences, especially for comparing two populations or a single population over time. We have unified social and natural sciences by showing that the above methods are applicable in both disciplines, at least if willing to take the trivial step of computing diversity as one minus segregation. Almost a decade ago, Reardon & Firebaugh [8] provided a unifying framework by which a suite of multi-group two-way segregation indices can be computed, each index being based on a different functional form for an association index. We extended their framework from two-way to multi-way tables. The main application that we outlined here was a segregation index that incorporates three variables – race, sex/gender, and company/department/industry – that could accommodate people selecting multiple races, multiple genders (transgender), and multiple employers (especially for part-time employment). We also provided examples showing how useful this is at measuring biological diversity. By using resampling techniques, social and natural scientists can use our extensions of Reardon & Firebaugh [8] to perform hypothesis tests of whether two populations have different indices of segregation or diversity.
